# Systemic Lipopolysaccharide-Induced Pain Sensitivity and Spinal Inflammation Were Reduced by Minocycline in Neonatal Rats

**DOI:** 10.3390/ijms19102947

**Published:** 2018-09-27

**Authors:** Cheng-Ta Hsieh, Yih-Jing Lee, Xiaoli Dai, Norma Beatriz Ojeda, Hyun Joon Lee, Lu-Tai Tien, Lir-Wan Fan

**Affiliations:** 1 School of Medicine, Fu Jen Catholic University, New Taipei City 24205, Taiwan; nogor@mail2000.com.tw (C.-T.H.); yjlee@mail.fju.edu.tw (Y.-J.L.); 2 Division of Neurosurgery, Department of Surgery, Cathay General Hospital, Taipei 10630, Taiwan; 3 Department of Chemistry, Fu Jen Catholic University, New Taipei City 24205, Taiwan; 4 Graduate Institute of Biomedical and Pharmaceutical Science, Fu Jen Catholic University, New Taipei City 24205, Taiwan; 5 Department of Anesthesiology, University of Mississippi Medical Center, Jackson, MS 39216, USA; xdai@umc.edu; 6 Department of Pediatrics, Division of Newborn Medicine, University of Mississippi Medical Center, Jackson, MS 39216, USA; nojeda@umc.edu; 7 Department of Neurobiology and Anatomical Sciences, University of Mississippi Medical Center, Jackson, MS 39216, USA; hlee@umc.edu; 8 Research Services, G.V. (Sonny) Montgomery Veterans Administration Medical Center, Jackson, MS 39216, USA

**Keywords:** minocycline, lipopolysaccharide, hyperalgesia, allodynia, spinal cord inflammation

## Abstract

In this study, we investigated the effects of minocycline, a putative suppressor of microglial activation, on systemic lipopolysaccharide (LPS)-induced spinal cord inflammation, allodynia, and hyperalgesia in neonatal rats. Intraperitoneal (i.p.) injection of LPS (2 mg/kg) or sterile saline was performed in postnatal day 5 (P5) rat pups and minocycline (45 mg/kg) or vehicle (phosphate buffer saline; PBS) was administered (i.p.) 5 min after LPS injection. The von Frey filament and tail-flick tests were performed to determine mechanical allodynia (a painful sensation caused by innocuous stimuli, e.g., light touch) and thermal hyperalgesia (a condition of altered perception of temperature), respectively, and spinal cord inflammation was examined 24 h after the administration of drugs. Systemic LPS administration resulted in a reduction of tactile threshold in the von Frey filament tests and pain response latency in the tail-flick test of neonatal rats. The levels of microglia and astrocyte activation, pro-inflammatory cytokine interleukin-1β (IL-1β), cyclooxygenase-2 (COX-2), and prostaglandin E2 (PGE2) in the spinal cord of neonatal rats were increased 24 h after the administration of LPS. Treatment with minocycline significantly attenuated LPS-induced allodynia, hyperalgesia, the increase in spinal cord microglia, and astrocyte activation, and elevated levels of IL-1β, COX-2, and PGE2 in neonatal rats. These results suggest that minocycline provides protection against neonatal systemic LPS exposure-induced enhanced pain sensitivity (allodynia and hyperalgesia), and that the protective effects may be associated with its ability to attenuate LPS-induced microglia activation, and the levels of IL-1β, COX-2, and PGE2 in the spinal cord of neonatal rats.

## 1. Introduction

Neonatal pain experiences and inflammation may possibly induce long-lasting alteration in pain sensitivity in both animal models and humans [1,2,3,4], and the early onset inflammatory hyperalgesia may last into adulthood [5,6,7]. Therefore, the treatment or prevention of further neurological damage induced by inflammation during infancy may be able to effectively reduce the incidence of pain.

In our previous studies, we developed a neonatal rat model to mimic the scenario of infection/inflammation through intracerebral (i.c.) or intraperitoneal (i.p.) injection of LPS in the postnatal day 5 (P5) rat brain [3,8]. In these models, we found that a low-dose injection of LPS resulted in brain injury and induced a great increase in microglia activation and interleukin-1β (IL-1β) concentrations in the neonatal rat brain [3]. Additional studies indicated that the administration of IL-1 or lipopolysaccharide (LPS) produced hyperalgesia (pain facilitation), which is possibly mediated by the induction of prostaglandin E2 (PGE2) [4,9,10,11]. However, the relationship between hyperalgesia and microglia activation following systemic inflammation in neonatal animals is unclear.

Evidence shows that a method that is used for the suppression of microglia activation has been developed with the potential for the treatment of pain [12,13]. It has been reported that minocycline (7-dimethylamino-6-dimethyl-6-deoxytetracycline), a suppressor of microglia, can penetrate into the central nervous system [14], and has an anti-inflammatory effect on the inhibition of microglial activation via the blocking of inflammatory cytokine secretion [15] and PGE2 production [16], which may have a potential effectiveness in the treatment of neuroinflammation [17,18] and neurodegenerative diseases [14,19,20]. A previous study revealed that minocycline prevented LPS-induced microglia cells and/or macrophage activation and cytokine response in the spinal cord and dorsal root ganglion in adult rats [21]. Thus, minocycline may prevent neuroinflammatory pain induced by LPS. However, the mechanism of minocycline’s effect on LPS-induced pain sensitivity needs to be further studied.

Our previous data indicated that neonatal i.c. LPS-injection resulted in hyperalgesia 24 h after drug administration and continued to 48 h or 72 h in rats. In addition, this hyperalgesia induced by i.c. LPS can be reduced by interleukin-1 receptor antagonist (IL-1ra) [3]. However, most of the routes of inflammation cases are via the peripheral area. Our previous study found that i.p. LPS shows the same tendency of white matter injury [8]. Therefore, in the present study, we chose the route of i.p. injection LPS in neonatal rats, which is a more pathophysiologically relevant approach. The objectives of this study are to determine (1) the protective effect of minocycline on hyperalgesia, and (2) the possible inflammatory factors through which minocycline provides protective effects against following neonatal LPS-induced spinal cord inflammation.

## 2. Results

### 2.1. Minocycline Attenuated Systemic LPS-Induced Mechanical Allodynia and Thermal Hyperalgesia

**Figure 1:**
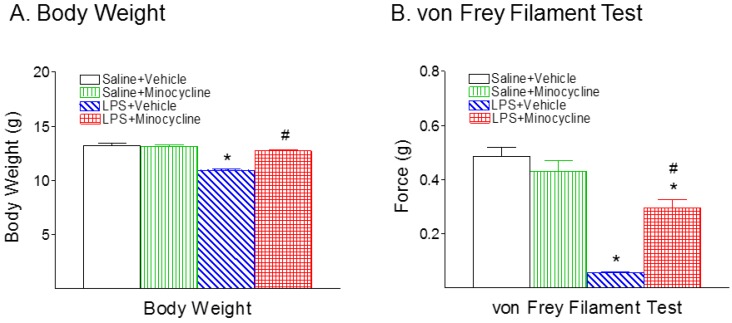
Minocycline attenuated systemic lipopolysaccharide (LPS)-induced body weight loss (**A**), and allodynia in the von Frey filament test (**B**) in the neonatal postnatal day 6 (P6) rats. (**A**), Systemic LPS treatment (P5) resulted in body weight loss in the P6 rats. Treatment with minocycline significantly reduced the LPS-induced body weight loss in the P6 rats. (**B**), Systemic LPS treatment (P5) resulted in a reduction of the withdrawal threshold (g force) for hind paw removal from a mechanical stimulation in the P6 rats. Treatment with minocycline significantly reduced LPS-induced pain hypersensitivity in the neonatal rats. The results are expressed as the mean ± SEM of 16 animals in each group. The results from body weight were analyzed by two-way ANOVA, followed by the Student–Newman–Keuls test. The results from the von Frey filament test were analyzed by Kruskal–Wallis one-way-ANOVA on ranks, followed by the Dunn’s test. * *p* < 0.05 represents a significant difference for the LPS + Vehicle group, or LPS + Minocycline group compared with the Saline + Vehicle group. ^#^ *p* < 0.05 represents a significant difference for the LPS + Minocycline group compared with the LPS + Vehicle group. (*n* = 16).

No significant difference was observed between the male and female rats within the same treatment group at P6. Therefore, data from rats of both sexes were combined and presented here. Consistent with our previous study, systemic LPS injection on P5 rats resulted in a lower body weight at P6 compared with the control group (*p* < 0.05) (Figure 1A). Minocycline treatment reduced the LPS-induced weight reduction in P6 rats (*p* < 0.05) (Figure 1A).

Mechanical allodynia was assessed by using the von Frey filament test. Systemic LPS injection decreased the pain threshold in the P6 rats compared with rat pups in the control group (*p* < 0.05) (Figure 1B). Minocycline treatment significantly reduced the LPS-induced mechanical allodynia in P6 rats (*p* < 0.05) (Figure 1B).

**Figure 2:**
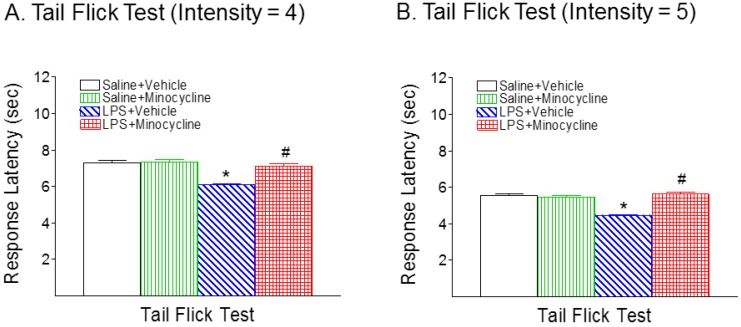
Minocycline attenuated systemic LPS-induced hyperalgesia in neonatal rats in the tail-flick test. Systemic LPS treatment (P5) resulted in reduction of mean latency times (second) in tail removal from a thermal stimulation ((**A**), intensity = 4; (**B**), intensity = 5) in the P6 rats. Treatment with minocycline significantly reduced LPS-induced pain hypersensitivity in the neonatal rats. The results are expressed as the mean ± SEM of 16 animals in each group and were analyzed by Kruskal–Wallis one-way ANOVA on ranks, followed by the Dunn’s test. * *p* < 0.05 represents significant difference for the LPS + Vehicle group compared with the Saline + Vehicle group. ^#^ *p* < 0.05 represents significant difference for the LPS + Minocycline group compared with the LPS + Vehicle group. (*n* = 16).

Figure 2 shows the effect of systemic LPS exposure on latency time in the tail-flick test in the rat pups, with or without minocycline. Rat pups in the LPS + Vehicle group showed a significant decrease in the mean latency in the tail-flick test compared with rat pups in the Saline + Vehicle control group (*p* < 0.05) with different thermal stimulation (Figure 2A, intensity = 4; and Figure 2B, intensity = 5). This decreased removal latency (i.e., an enhanced response to stimuli) is characteristic of hyperalgesia in the neonatal rats. Minocycline treatment effectively prevented systemic LPS-induced pain hypersensitivity in neonatal rats (*p* < 0.05) (Figure 2A,B).

### 2.2. Minocycline Attenuated Systemic LPS-Induced Increase in Microglia Activation and Inflammatory Responses in the Spinal Cord

**Figure 3:**
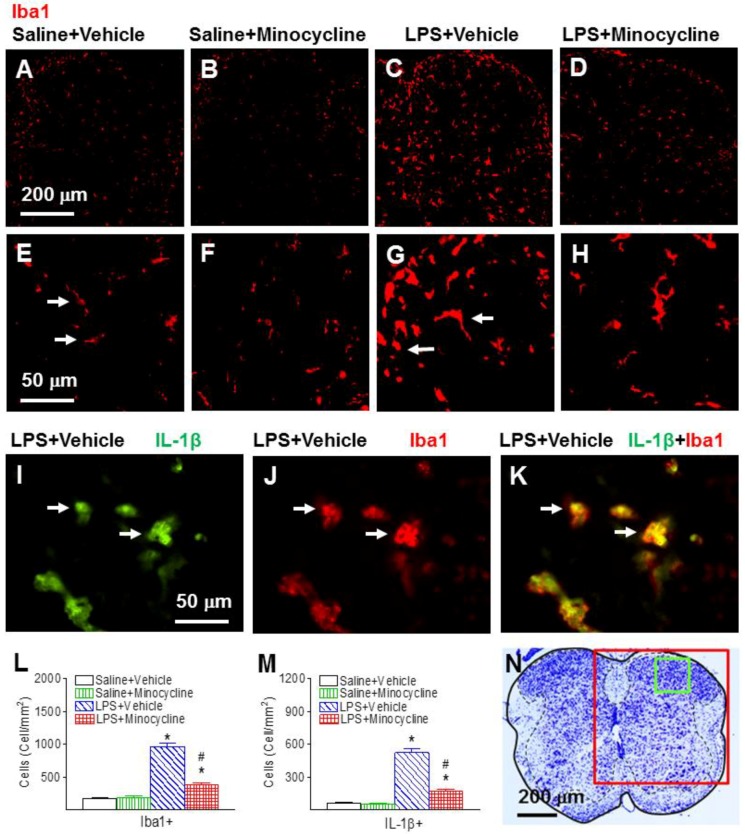
Minocycline reduced neonatal LPS-induced microglia activation, as assessed by ionized calcium binding adapter molecule 1 (Iba1+) staining in the spinal dorsal horn of the P6 rats. The scheme used a Nissl staining section (**N**) presenting an overview over the detected region: red box, (**A**–**D**); green box, (**E**–**K**). Representative photomicrographs of Iba1 immunostaining (**A**–**H**) in the rat spinal cord 24 h (P6) after LPS injection. Most of the microglia were in a resting state with a ramified shape (arrows indicated in (**E**)) in the rat spinal dorsal horn of the Saline + Vehicle group (**A**,**E**). LPS exposure induced the increase of numerous activated microglia with enlarged cell bodies and blunt processes ((**C**,**G**), arrows indicated in (**G**)). Minocycline attenuated an LPS-induced increase in activated microglia (**D**). Microglia activation was quantified by counting the number of Iba1+ cells in the spinal dorsal horn (**L**). Minocycline also reduced neonatal LPS-induced interleukin-1β (IL-1β) expressing cells in the spinal dorsal horn of the P6 rat (**M**). Double-labeling showed that many Iba1+ activated microglia in the spinal dorsal horn ((**J**), red) of the LPS-injected rat brain were IL-1β-expressing cells ((**I**), green). **K** (yellow) is a merged image of (**I**,**J**). IL-1β expression was quantified by counting the number of IL-1β+ cells in the spinal dorsal horn (**M**). The results are expressed as the mean ± SEM of eight animals in each group and analyzed by two-way ANOVA, followed by the Student–Newman–Keuls test. * *p* < 0.05 represents a significant difference for the LPS + Vehicle group, or LPS + Minocycline group compared with the Saline + Vehicle group. ^#^ *p* < 0.05 represents a significant difference for the LPS + Minocycline group compared with the LPS + Vehicle group. (*n* = 8).

Systemic LPS treatment stimulated the activation of microglia in the spinal cord as indicated by ionized calcium binding adapter molecule 1 (Iba1+) immunostaining, which was determined in the lumbar spinal cord sections between L4 and L5 in the P6 rats (Figure 3). In the control rat brain (Figure 3A,E), a few Iba1+ cells were detected, and most of those cells were in resting status with a ramified shape (arrows indicated in Figure 3E). Significantly increased numbers of activated microglia showed bright staining of an elongated or a round-shaped cell body with blunt or no processes, which is an indication of microgliosis (arrows indicated in Figure 3G) that was found in the spinal dorsal horn of the rat 24 h after LPS injection (*p* < 0.05) (Figure 3C,G,L). Minocycline treatment reduced the systemic LPS-induced increase in the number of activated microglia in the spinal dorsal horn of P6 rats (*p* < 0.05) (Figure 3D,H,L).

**Figure 4:**
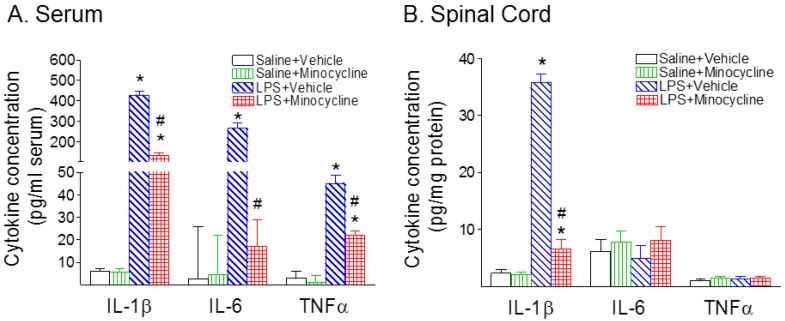
Minocycline attenuated systemic LPS exposure-induced increases in inflammatory cytokines (IL-1β, interleukin-6 (IL-6), and tumor necrosis factor-α (TNF-α)) in the rat serum (**A**) and spinal cord (**B**) 24 h (P6) after LPS injection. (**A**), The concentration levels of IL-1β, IL-6, and TNF-α 24 h (P6) following LPS injection in the serum were elevated compared with the control group. Minocycline attenuated an LPS-induced increase in the concentration levels of IL-1β, IL-6, and TNF-α in the serum of P6 rats. (**B**), Following LPS injection, the concentration of IL-1β in the spinal cord was elevated compared with the control group in the P6 rats. Minocycline attenuated LPS-induced increase in the concentration levels of IL-1β in the spinal cord of P6 rats. The results are expressed as the mean ± SEM of eight animals in each group and analyzed by two-way ANOVA, followed by the Student–Newman–Keuls test. * *p* < 0.05 represents a significant difference for the LPS + Vehicle group, or LPS + Minocycline group compared with the Saline + Vehicle group. ^#^ *p* < 0.05 represents significant difference for the LPS + Minocycline group compared with the LPS + Vehicle group. (*n* = 8).

Systemic exposure to LPS resulted in inflammatory responses in the rat serum and spinal cord, as evidenced by the increase in IL-1β+ cells (Figure 3I,K) and the elevation of pro-inflammatory cytokine levels (Figure 4A, serum; and Figure 4B, spinal cord). Double-labeling showed that many of the activated microglia (Iba1+) were IL-1β-expressing cells (Figure 3I–K). There were few IL-1β+ cells that were glial fibrillary acidic protein (GFAP)-expressing astrocytes (data not shown). Twenty-four hours (P6) following LPS injection, IL-1β, interleukin-6 (IL-6), and tumor necrosis factor-α (TNFα) concentrations in the serum of LPS-exposed rats were dramatically increased compared to those in the saline-injected rats (*p* < 0.05) (Figure 4A). Minocycline attenuated an LPS-induced increase in the concentration levels of IL-1β, IL-6, and TNF-α in the serum of P6 rats (*p* < 0.05) (Figure 4A). IL-1β concentrations in the LPS-exposed rat spinal cord were also significantly increased as compared to those in the control rat spinal cord (*p* < 0.05) (Figure 4B). Minocycline attenuated an LPS-induced increase in the concentration levels of IL-1β in the spinal cord of P6 rats (*p* < 0.05) (Figure 4B).

### 2.3. Minocycline Attenuated Systemic LPS-Induced Increase in Astrocyte Activation, COX-2, and PGE2 Expression in the Spinal Cord

**Figure 5:**
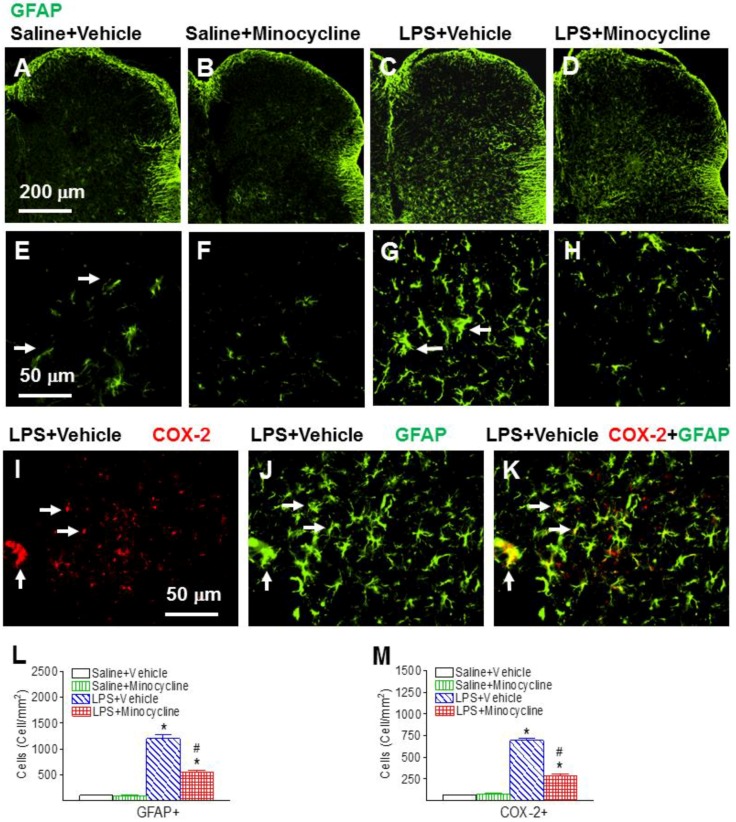
Minocycline reduced neonatal LPS-induced astrocyte activation, as assessed by glial fibrillary acidic protein (GFAP+) staining in the spinal dorsal horn of the P6 rats. Representative photomicrographs of GFAP immunostaining (**A**–**H**) in the rat spinal cord 24 h (P6) after LPS injection. Most of the astrocytes were in a resting state with fine processes extending from the main cellular processes (arrows indicated in (**E**)) in the rat spinal dorsal horn of the Saline + Vehicle group (**A**,**E**). LPS exposure induced the increase of numerous activated astrocytes with the hypertrophy of cellular processes ((**C**,**G**), arrows indicated in (**G**)). Minocycline attenuated an LPS-induced increase in activated astrocytes (**D**). Astrocyte activation was quantified by counting the number of GFAP+ cells in the spinal dorsal horn (**L**). Minocycline also reduced neonatal LPS-induced cyclooxygenase-2 (COX-2) expressing cells in the spinal dorsal horn of the P6 rat (**M**). Double-labeling showed that many GFAP+ activated microglia in the spinal dorsal horn ((**J**), green) of the LPS-injected rat brain were COX-2 expressing cells ((**I**), red). **K** (yellow) is a merged image of (**I**,**J**). COX-2 activation was quantified by counting the number of COX-2+ cells in the spinal dorsal horn (**M**). The results are expressed as the mean ± SEM of eight animals in each group and analyzed by two-way ANOVA, followed by the Student–Newman–Keuls test. * *p* < 0.05 represents a significant difference for the LPS + Vehicle group, or LPS + Minocycline group compared with the Saline + Vehicle group. ^#^ *p* < 0.05 represents significant difference for the LPS + Minocycline group compared with the LPS + Vehicle group. (*n* = 8).

The effects of systemic LPS exposure on the spinal astrocytes, as indicated by GFAP+ immunostaining, were determined in the P6 rat lumbar spinal cord (Figure 5). Most of the astrocytes were in a resting state with fine processes extending from the main cellular processes (arrows indicated in Figure 5E) in the rat spinal dorsal horn of the control group (Figure 5A,E). Systemic LPS exposure induced the increase of numerous activated astrocytes (GFAP+ cells) with a hypertrophy of cellular processes (arrows indicated in Figure 5G), which is an indication of astrogliosis, in the P6 rat spinal cord (*p* < 0.05) (Figure 5C,G,L). Minocycline reduced the number of activated astrocytes in the rat spinal dorsal horn following systemic LPS injection (*p* < 0.05) (Figure 5D,H,L).

**Figure 6:**
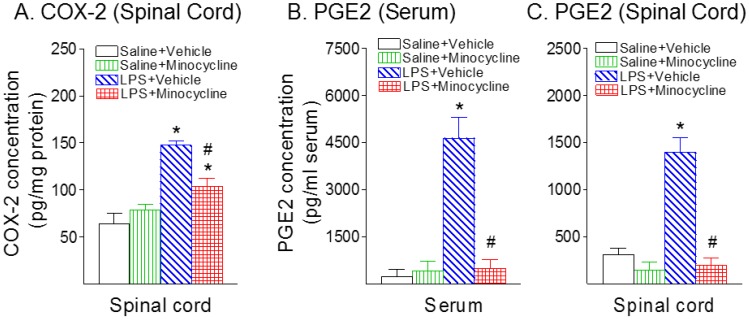
Minocycline attenuated systemic LPS exposure-induced increases in cyclooxygenase-2 (COX-2) in the rat spinal cord (**A**), and prostaglandin E2 (PGE2) in the rat serum (**B**) and spinal cord (**C**) 24 h (P6) after LPS injection. (**A**), Following LPS injection, the concentration of COX-2 in the spinal cord was elevated compared with the control group in the P6 rats. Minocycline attenuated an LPS-induced increase in the concentration levels of COX-2 in the spinal cord of P6 rats. (**B**,**C**), The concentration levels of PGE2 24 h (P6) following LPS injection in the serum (**B**) and spinal cord (**C**) were elevated compared with the control group. Minocycline attenuated an LPS-induced increase in the concentration levels of PGE2 in the serum and spinal cord of P6 rats. The results are expressed as the mean ± SEM of eight animals in each group and analyzed by two-way ANOVA, followed by the Student–Newman–Keuls test. * *p* < 0.05 represents a significant difference for the LPS + Vehicle group compared with the Saline + Vehicle group. ^#^ *p* < 0.05 represents a significant difference for the LPS + Minocycline group compared with the LPS + Vehicle group. (*n* = 8).

Neonatal systemic LPS-induced inflammatory responses also can be indicated by the elevation of COX-2 expression levels, which is induced in inflammatory cells in response to cytokines and pro-inflammatory molecules [22,23]. Following LPS injection, the COX-2+ staining cell number (Figure 5I,M) and the concentration of COX-2 in the spinal cord was elevated compared with the control group in the P6 rats (*p* < 0.05) (Figure 6A). Minocycline reduced an LPS-induced increase in the COX-2+ staining cell number (*p* < 0.05) (Figure 5M) and the concentration levels of COX-2 in the spinal cord of P6 rats (*p* < 0.05) (Figure 6A). Double-labeling showed that most of the COX-2+ cells in the spinal dorsal horn were GFAP+ cells (Figure 5I–K). There were few COX-2+ cells that were Iba1-expressing microglia (data not shown).

The key pain mediator PGE2, which is produced by COX-2 [24], was determined in rat serum and spinal cord following systemic LPS exposure (Figure 6B, serum; Figure 6C, spinal cord). The concentration levels of PGE2 24 h (P6) following LPS injection in the serum (Figure 6B) and spinal cord (Figure 6C) were elevated compared with the control group (*p* < 0.05). Minocycline attenuated an LPS-induced increase in the concentration levels of PGE2 in the serum and spinal cord of P6 rats (*p* < 0.05) (Figure 6B,C).

## 3. Discussion

Neonatal pain is poorly rarely understood and is often undertreated, especially in preterm infants who were exposed to inflammation or invasive medical procedures during neonatal intensive care treatment [5,25,26]. However, the neonatal pain experiences may increase subsequent pain sensitivity (hyperalgesia) by disrupting pain processing and altering nociceptive neuronal circuits [3,27,28], and the early onset inflammatory hyperalgesia may last into adulthood [5,6,7]. It is important to find ways to treat or prevent the pain sensitivity induced by inflammation during infancy, which may be able to effectively reduce the incidence of pain in later life.

**Figure 7:**
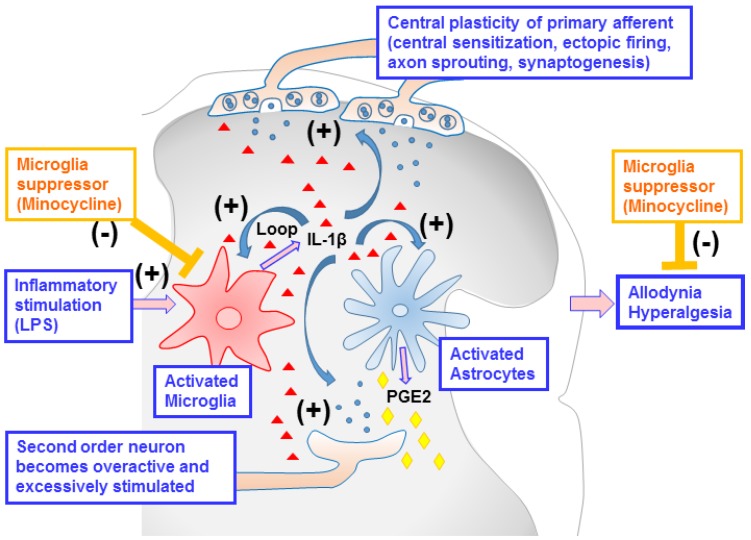
Schematic illustration of glia–glia and neuron–glia interaction in the spinal cord dorsal horn in neonatal systemic inflammation-induced pain hypersensitivity. Systemic LPS exposure led to spinal cord inflammation, including increased microglia and astrocyte activation, microglia-related pro-inflammatory cytokine interleukin-1β (IL-1β), and pain mediator prostaglandin E2 (PGE2), which induced a positive feedback loop. The above effects may cause primary afferent neurons to undergo central plasticity (central sensitization, ectopic firing, axon sprouting, synaptogenesis), and second-order neurons to become overactive and excessively stimulated in the spinal cord dorsal horn, which enlarges the effect of original insults on nociception and results in the enhancement of pain sensitivity (allodynia and hyperalgesia) in neonatal rats. These compromises in both gliosis and behaviors are attenuated by minocycline (a microglia suppressor), suggesting that microglia activation plays a critical role in mediating these pathological changes.

Our data showed that systemic LPS injections induced mechanical allodynia and thermal hyperalgesia in neonatal rats. These pain responses in hindlimbs and tails may result from the hypersensitivity of nociceptive afferents in the lumbosacral spinal cord. It is well established in adult rodents that systemic inflammatory responses enrich neurotrophic factor e.g., nerve growth factor (NGF) in the dorsal root ganglia (DRG) and in the spinal cord, which activates the central plasticity of nociceptive afferents [29]. The mechanisms of the central plasticity that give rise to nociceptive hypersensitivity include changes of function, e.g., central sensitization, ectopic firing, and changes of structure, e.g., axon sprouting, synaptogenesis [30], as summarized in Figure 7. Nociceptive afferents in neonatal animals seem to share the mechanisms of the central plasticity with those afferents in adults, but the temporal patterns may differ depending on postnatal ages. For instance, C fibers bearing calcitonin gene-related peptide (CGRP) underwent central sprouting in laminae I and II starting from P6 when complete Freund’s adjuvant (CFA) was injected as early as P1 [31]. This supports the current results showing that minocycline treatments alleviated pain behaviors—both mechanical allodynia and thermal hyperalgesia—induced by systemic LPS injections at P6. Collectively, blocking systemic inflammation with a microglia suppressor, minocycline, might neutralize the central plasticity of nociceptive afferents in the lumbosacral dorsal horn and, as a result, prevent the development of pain sensitivity in postnatal rats.

Neuroinflammatory response in the spinal dorsal horn involves a complex interaction between neurons and glial cells, and leads to the release of inflammatory mediators and the induction of a positive feedback loop that enlarges the effect of original insults on nociception [32,33]. The present results showed that hyperalgesia and allodynia induced by systemic inflammation are closely associated with increases in the microglia and astrocyte activation, as well as microglia-related pro-inflammatory cytokines IL-1β, COX-2, and COX-2 product PGE2 in the P6 spinal cord. During the development of hyperalgesia, the activation of the microglia is hypothesized to play an important role in regulating spinal cord inflammation [34,35]. This response may be initiated by microglia and amplified by astrocytes, the major glial cell population in the central nervous system [35,36,37]. Many mediators are known to activate the microglia, including cytokines, chemokines, adenosine triphosphate, glutamate, and neuropeptides [38]. Activated microglia subsequently release inflammatory cytokines, including IL-1β, IL-6, IL-10, brain-derived neurotropic factor (BDNF), TNF and tumor growth factor-β (TGF-β), which may trigger the hyperalgesia. The treatment of minocycline prevents neonatal systemic LPS exposure-induced pain hypersensitivity (allodynia and hyperalgesia), and the preventive effects may be associated with its ability to attenuate LPS-induced microglia activation, and microglia-related pro-inflammatory cytokine IL-1β and pain mediator PGE2. The manipulation of microglia-mediated neuroinflammation by minocycline treatments provides a mechanistic approach for the prevention of inflammatory hyperalgesia, and could be developed as an effective treatment for pain hypersensitivity.

The occurrence of maternal or placental infection is frequently associated with increased concentrations of inflammatory cytokines such as tumor necrosis factor-α (TNF-α), IL-1β, and IL-6 in the infant brain [39,40]. The role of IL-1β has been implicated in the modulation of pain sensitivity and mediating the hyperalgesia and allodynia produced by LPS-induced inflammation [41]. The intrathecal administration of IL-1β also induced mechanical allodynia and thermal hyperalgesia [42], and treatments with IL-1 receptor antagonist (IL-1ra) could inhibit hyperalgesic responses to LPS, IL-1β, carrageenin bradykinin, and TNF-α [41]. There is evidence in mice models that impaired IL-1 signaling or chronic treatment with IL-1ra alone has resulted in reduced pain responses even under non-inflammatory states [11], which suggests a direct role of IL-1 for pain sensitivity.

IL-1β has been discovered to be an important inflammatory factor in mediating mechanical or thermal hyperalgesia [43,44,45]. IL-1 also stimulates the production of arachidonic acid metabolites, especially PGE-2, through up-regulating the expression of COX-2 [41,44,46]. PGE2 has been indicated as having a pro-nociceptive role in pain signal processing in the peripheral nervous system and the spinal cord, which was implicated in neuroinflammatory pain, neuropathic pain, visceral pain, and migraine headache [24]. Furthermore, blocking IL-1 signaling has been found to ameliorate intracerebral LPS-induced long-lasting hyperalgesia in neonatal rats [3]. As previously shown, the systemic injection of LPS in adult rats significantly increased mRNA expressions levels only for IL-1β, TNFα, monocyte chemotactic protein 1, and RANTES (regulated on activation, normal, T-cell expressed and secreted, alternative name CCL5 (C-C chemokine ligand 5)), but not for IL-6 in the spinal cord [21]. In neonatal rats, our present studies revealed that the systemic injection of LPS induced the increases in the expressions of IL-1β, IL-6, TNFα, and PGE2 proteins in the serum. However, only the expressions of the IL-1β and PGE2 proteins were significantly increased in the spinal cord of P6 rats. These results were similar to the data from our and other laboratories showing that the levels of IL-6 or TNFα were greatest 3 h to 6 h post LPS, but were returned to baseline by 24 h post LPS in the spinal cord [47] or brain [48,49]. It has been reported that LPS is often administered systemically to induce central nervous system (CNS) inflammation, as it does not cross the blood–brain barrier [50,51]. Potential molecules crossing the blood–brain barrier to trigger CNS inflammatory activities include the cytokines (e.g., IL-1β and TNF-α) [47,52] or PGEs produced by perivascular macrophages and/or endothelial cells that line the blood–brain barrier [53,54,55]. However, the detailed mechanisms of CNS inflammation induced by systemic LPS injection need to be further studied. Based on these findings, our data suggest the role of IL-1β rather than IL-6 or TNFα for the regulation of PGE2 that mediates LPS-induced inflammatory reactions both at the systemic level and in the central nervous system in our animal model.

Minocycline has antibiotic effects and multiple non-antibiotic effects, and is commonly used in many animal models of neuropathic pain, neurodegenerative disorders, neuroinflammatory conditions, or ischemic injury [3,17,18,20,56]. Our previous study indicated that treatment with minocycline significantly attenuated the LPS-induced brain injury and improved the sensorimotor neurobehavioral performance, including the righting reflex and wire hanging maneuver in neonatal rats [18]. Similar with our previous finding, minocycline treatment attenuated LPS-induced body weight loss, which has been attributed to the combination of protein loss (TNFα-induced activation of ubiquitin–proteosome system-mediated muscle proteolysis) [57,58,59], increased substrate utilization, reduced intake, and diarrhea with associated fluid loss [59]. It has also been previously reported that minocycline treatment reduces LPS-induced hyperalgesia and allodynia in adult rodents [21,60,61]. Yoon et al. indicated that minocycline blocks LPS-induced hyperalgesia by the suppression of microglia but not astrocytes, which differed from our study. A possible reason is the difference in the timing of LPS treatment between the previous study, which used adult rats [21], and ours, which used postnatal day five (P5) rats. Minocycline not only inhibits the proliferation and activation of microglia, leading to impairment in the production of inflammatory and pain mediators [62,63], but also influences the process of apoptosis [64] and promotes the generation of dendritic cells [45]. Our previous studies demonstrated that minocycline treatment attenuated cerebral white matter injury and reduced neurological impairment in neonatal rats following intracerebral LPS injection [17,18]. These findings suggest the potential development of an effective therapeutic treatment with minocycline. However, chronic treatment with minocycline may produce unintended side effects. For instance, an in vitro study found that minocycline negatively impacts immune cell subsets, such as T cells, which may cause increased susceptibility to CNS infection [13,65]. Thus, the chronic minocycline treatment’s effect on the chronic pain hypersensitivity should be further studied.

Clinical and animal research studies indicate that the maturation of nociceptive circuitry occurs in the absence of adequate stimuli during the perinatal period, and that perinatal exposure to noxious stimulation can lead to long-term alteration in subsequent physiological and behavioral responses to somatosensory stimulation [4,5,6]. The current results indicate that LPS-induced systemic inflammation causes the enhancement of pain sensitivity in neonatal rats, and treatment with microglia suppressor minocycline protects against microglia activation-related spinal inflammation and pain hypersensitivity. Our results demonstrate that neonatal treatment with minocycline immediately after the inflammatory stimulation can reduce inflammation and pain behavioral responses to prevent these phenomena from carrying on into the adulthood.

## 4. Materials and Methods

### 4.1. Chemicals

Unless otherwise stated, all of the chemicals used in this study were purchased from Sigma (St. Louis, MO, USA). Monoclonal mouse antibodies against glial fibrillary acidic protein (GFAP), and polyclonal rabbit antibodies against ionized calcium binding adapter molecule 1 (Iba1) were purchased from Millipore (Billerica, MA, USA) and Wako Chemicals USA (Irvine, CA, USA), respectively. Polyclonal goat antibody against interlukin-1β (IL-1β) and cyclooxygenase-2 (COX-2) were obtained from Novus Biologicals (Littleton, CO, USA) and Santa Cruz Biotechnology (Santa Cruz, CA, USA), respectively. ELISA kits for immunoassays of rat IL-1β, interleukin-6 (IL-6), tumor necrosis factor-α (TNFα), prostaglandin E2 (PGE-2), and COX-2 were purchased from R&D Systems (Minneapolis, MN, USA), and NeoScientific (Cambridge, MA, USA), respectively.

### 4.2. Animals

Timed pregnant Sprague–Dawley rats arrived in the laboratory on day 19 of gestation. Animals were maintained in an animal room on a 12-h light/dark cycle and at constant temperature (22 ± 2 °C). The day of birth was defined as postnatal day 0 (P0). After birth, the litter size was adjusted to 10 pups per litter to minimize the effect of litter size on body weight and spinal cord size. All of the procedures for animal care were approved by the Institutional Animal Care and Use Committee at the University of Mississippi Medical Center (0834D; approval on 17 July 2012; 0834E; approval on 5 February 2015; 0834F; approval on 5 January 2018) or Fu Jen Catholic University (A10310; approval on 1 June 2014). Every effort was made to minimize the number of animals used and their suffering.

### 4.3. Animal Treatment

Rat pups were divided into four groups: Saline + Vehicle (*n* = 16), Saline + Minocycline (*n* = 16), LPS + Vehicle (*n* = 16), and LPS + Minocycline (*n* = 16). Intraperitoneal injection of LPS (2 mg/kg, from *Escherichia coli*, serotype 055: B5) or sterile saline (total volume of 0.1 mL) in five-day-old Sprague–Dawley rat pups of both sexes was performed as previously described [17,18]. Then, 45 mg/kg of minocycline in phosphate buffer saline (PBS) or PBS alone with a total volume of 0.1 mL was injected 5 min after the LPS injection [17]. Twenty-four hours after the injection, 16 pups were included in each treatment group for body weight record and behavioral tests. Eight pups from each group were sacrificed by decapitation for fresh lumbar spinal cord tissue collection. Another eight pups from each group were sacrificed by transcardiac perfusion with normal saline followed by 4% paraformaldehyde for spinal cord section preparation. Consecutive frozen lumbar spinal cord sections (the lumbar enlargement of the spinal cord, L4~L5) at a thickness of 10 µm were prepared in a cryostat for immunohistochemistry examination.

### 4.4. Von Frey Filament Test

This test is used to assess the mechanical nociception (cutaneous mechanical sensitivity) of rats. Each rat was placed individually under an inverted acrylic box on raised wire mesh (Dynamic Plantar Anesthesiometer, UGO BASILE, Varese, Italy) and was allowed to acclimatize to the new environment for 5 min. Mechanical thresholds for the flexion withdrawal reflexes in response to punctuating the mechanical stimulation of the surface of the rat’s hind paw were tested using von Frey filaments that exert a reproducible stimulus strength in grams (ranging from 0.236 to 0.384 mm in diameter with marking forces of 0.02–0.60 g). Filaments were applied five times at 10-s intervals alternately to the plantar surface of one hind paw, and five times at 10-s intervals alternately to the other hind paw after a 3-min waiting period. The response threshold is defined as the von Frey filament that produced reflex paw withdrawal in three out of five applications [66,67].

### 4.5. Tail-Flick Test

The tail-flick test is designed to assess the thermal nociceptive threshold by using an infrared source. The tail-flick test was performed as described by our previous study with modifications at 24 h after LPS injection [3,68,69]. Rats were habituated to handling and being inserted into a plastic cylindrical tube. A shallow groove cut into the Plexiglas plate supported the rat’s tail during the trials of tests. A small section halfway from the tip to the base of the rat’s tail was placed under radiant heat (Analgesia Test Tail-Flick Type 812, Columbus Instruments, Columbus, OH, USA). The pain sensitivity was measured by tail-flick latency, which is defined as the time from the onset of radiant heat to tail withdrawal. A cut-off time of 10 s was set to prevent thermal injury. The tail-flick latency was calculated as the average of three tail-flick latencies.

### 4.6. Immunohistochemistry

Spinal cord inflammation was estimated based on the results of immunohistochemistry in consecutive lumbar spinal cord sections between L4 to L5 (~3 mm) at 10 μm of thickness prepared from rats sacrificed one day (P6) after LPS injection. Primary antibodies were used with the following dilutions: Iba1 (1:500), IL-1β (1:100), GFAP (1:200), and COX-2 (1:100). Microglia was detected using Iba1 immunostaining, which recognizes both resting and activated microglia. GFAP was used to detect astrocytes. COX-2 provides the selective staining of inducible cyclooxygenase. Sections were incubated with primary antibodies at 4 °C overnight and further incubated with secondary antibodies conjugated with fluorescent dyes (Alexa Fluor 555, 1:500 or Alexa Fluor 488, 1:200; Invitrogen, Carlsbad, CA, USA) for 1 h in the dark at room temperature. 4′,6-Diamidino-2-phenylindole (DAPI) (100 ng/mL) was used simultaneously to stain nuclei and aid in their identification during the final visualization. Sections incubated in the absence of primary antibody were used as negative controls. When double-labeling was required, primary antibodies from different hosts were used in combination with appropriate secondary antibodies, which were against the immunoglobulin from the corresponding hosts. The resulting sections were examined under a fluorescent microscope (Nikon Ni-E, Melville, NY, USA) at appropriate wavelengths.

### 4.7. Enzyme-Linked Immunosorbent Assay (ELISA)

Concentrations of IL-1β, IL-6, TNF-α, COX-2, and PGE2 in the rat spinal cord at 24 h after the LPS exposure were measured as markers of LPS-induced inflammatory responses by ELISA as previously described [8,48,70]. Briefly, serum and spinal cord tissues from each pup were collected 24 h after LPS injection, when the LPS-stimulated increase in inflammatory cytokines in the rat brain reached a peak value [49]. Spinal cord tissues were homogenized by sonication in 1 mL of ice-cold PBS (pH 7.2) and centrifuged at 12,000× *g* for 20 min at 4°C. The supernatant was collected, and the protein concentration was determined by the Bradford method. ELISA was performed according to the manufacturer’s instructions, and data were acquired using a 96-well plate reader (Bio-Tek instruments, Inc., Winooski, VT, USA). The cytokine contents were expressed as picograms of cytokines per milligram of protein.

### 4.8. Quantification of Data and Statistics

Spinal cord sections at the lumbar L4~L5 levels were used for the determination of microglia and astrocyte changes caused by systemic LPS injection. Immunostaining data were quantified by the counting of positively stained cells. When the cellular boundary was not clearly separated, numbers of DAPI-stained nuclei from the superimposed images were counted as the cell number. Three digital microscopic images were randomly captured in the spinal dorsal horn region in each of the three sections, and the number of positively stained cells in the three images was counted and averaged (cells/mm^2^). The mean value of cell counting from the three spinal cord sections was used to represent one single spinal cord. For the convenience of comparison among the treatment groups, results were standardized as the average number of cells/mm^2^.

Data from body weight, immunostaining, and ELISA assay were presented as the mean ± SEM and analyzed by two-way ANOVA followed by the Student–Newman–Keuls test. Data from the von Frey filament test and tail-flick test were analyzed by Kruskal–Wallis one-way ANOVA on ranks, followed by the Dunn’s test. Results with a *p* < 0.05 were considered statistically significant.

## 5. Conclusions

In summary, the current study demonstrates that systemic LPS exposure leads to spinal dorsal horn inflammation and the enhancement of pain sensitivity (allodynia and hyperalgesia) in neonatal rats. These compromises in both gliosis and behaviors are attenuated by minocycline (a microglia suppressor), and the preventive effects may be associated with its ability to attenuate LPS-induced microglia and astrocyte activation and microglia/astrocyte-related pro-inflammatory cytokines and pain mediators (Figure 7). Our findings suggest that microglia activation plays a critical role in mediating these pathological changes, and targeting microglia-mediated neuroinflammation may be a potential treatment for pain hypersensitivity caused by systemic inflammation in neonates.
